# The Spatial and Temporal Characterization of Gut Microbiota in Broilers

**DOI:** 10.3389/fvets.2021.712226

**Published:** 2021-08-30

**Authors:** Qianqian Zhou, Fangren Lan, Xiaochang Li, Wei Yan, Congjiao Sun, Junying Li, Ning Yang, Chaoliang Wen

**Affiliations:** National Engineering Laboratory for Animal Breeding and Key Laboratory of Animal Genetics, Breeding and Reproduction, Ministry of Agriculture and Rural Affairs, China Agricultural University, Beijing, China

**Keywords:** broiler, gut microbiota, spatial heterogeneity, temporal colonization, segment-related bacteria

## Abstract

The gut microbiota of chickens plays an important role in host physiology. However, the colonization and prevalence of gut microbiota have not been well-characterized. Here, we performed 16S rRNA gene sequencing on the duodenal, cecal and fecal microbiota of broilers at 1, 7, 21, and 35 days of age and characterized the dynamic succession of microbiota across the intestinal tract. Our results showed that Firmicutes was the most abundant phylum detected in each gut site at various ages, while the microbial diversity and composition varied among the duodenum, cecum, and feces at different ages. The microbial diversity and complexity of the cecal microbiota increased with age, gradually achieving stability at 21 days of age. As a specific genus in the cecum, *Clostridium_sensu_stricto_1* accounted for 83.50% of the total abundance at 1 day of age, but its relative abundance diminished with age. Regarding the feces, the highest alpha diversity was observed at 1 day of age, significantly separated from the alpha diversity of other ages. In addition, no significant differences were observed in the alpha diversity of duodenal samples among 7, 21, and 35 days of age. The predominant bacterium, *Lactobacillus*, was relatively low (0.68–6.04%) in the intestinal tract of 1-day-old chicks, whereas its abundance increased substantially at 7 days of age and was higher in the duodenum and feces. *Escherichia-Shigella*, another predominant bacterium in the chicken intestinal tract, was also found to be highly abundant in fecal samples, and the age-associated dynamic trend coincided with that of *Lactobacillus*. In addition, several genera, including *Blautia, Ruminiclostridium_5, Ruminococcaceae_UCG-014*, and *[Ruminococcus]_torques_group*, which are related to the production of short-chain fatty acids, were identified as biomarker bacteria of the cecum after 21 days of age. These findings shed direct light on the temporal and spatial dynamics of intestinal microbiota and provide new opportunities for the improvement of poultry health and production.

## Introduction

As a high-quality source of animal protein, chicken meat is an important component of a healthy and well-balanced diet for humans (1). The demand for chicken products has grown rapidly in recent decades (2). More than 72 billion broiler chickens were produced in 2019 (FAOSTAT), making chicken meat widely available and more affordable than other meats. With the global population approaching 8 billion people, ensuring an adequate supply of safe food has become increasingly important, especially for developing countries.

The intestinal microbiota is crucial for host health and productivity (3). Previous studies have demonstrated that specific gut microbiota was strongly linked to chicken phenotypes such as feed efficiency (4) and fat deposition (5). *Lactobacillus* strains inhabit the chicken gut microbiota and express antimicrobial activities that participate in the gastrointestinal tract (GIT) system of defense of the host (6, 7). *Salmonella* and *Campylobacter* contamination is highly prevalent in poultry production, and poultry is often implicated as a main source of human infection (8–11).

However, the microbial composition of the chicken GIT is not static but presents temporal variations related to age (12). Videnska et al. (13) suggested four distinct developmental phases of the cecal microbiota in egg-type chickens in their production cycle. In meat-type chickens, several studies revealed a succession of bacterial communities and an increasing microbial diversity in different compartments of the GIT during growth (14, 15). Newly hatched chicks with small amounts of bacteria are susceptible to environmental conditions, and the composition of their intestinal microbiota is largely dependent on the surrounding environment (16). The establishment of the gut microbiota occurs quickly and is primarily colonized by facultative anaerobes. The simple microbiota gradually transits to complex and obligate anaerobes with age and eventually reaches a relatively stable dynamic state (6, 12, 15).

In addition, the chicken GIT is composed of many different regions, and each region plays a unique role in nutrient digestion and absorption and harbors its own unique microbial composition (6, 17, 18). Chickens have two paired ceca, and both harbor similar bacterial communities (17). The cecum has attracted the most attention because of its high microbial density and metabolism-related functions, acting as a key region for bacterial fermentation of nondigestible carbohydrates (19). Most of the cecal microorganisms are obligate anaerobes, including *Clostridium, Bacteroides*, and *Ruminococcus* (20). The small intestine, including the duodenum, jejunum, and ileum, where nutrients are primarily digested and absorbed, contains lower numbers of microorganisms and tends to be colonized primarily by acid-tolerant and facultative anaerobes such as *Lactobacillus, Enterococcus*, and *Streptococcus* (21, 22). The composition of the fecal microbiota largely fluctuates depending on varying contributions of microbiota from different gut segments (23). Owing to the convenience and non-invasiveness of fecal sampling, feces is a common proxy for the gut microbial community.

Therefore, the objective of the present study was to compare the microbial composition of the duodenum, cecum and feces at four timepoints: 1, 7, 21, and 35 days of age. A detailed understanding of the spatial-temporal succession of the gut microbial composition could help to develop new interventions to optimize the gut microbiota that would ultimately improve production performance.

## Materials and Methods

### Animal and Sample Collection

**Figure 1:**
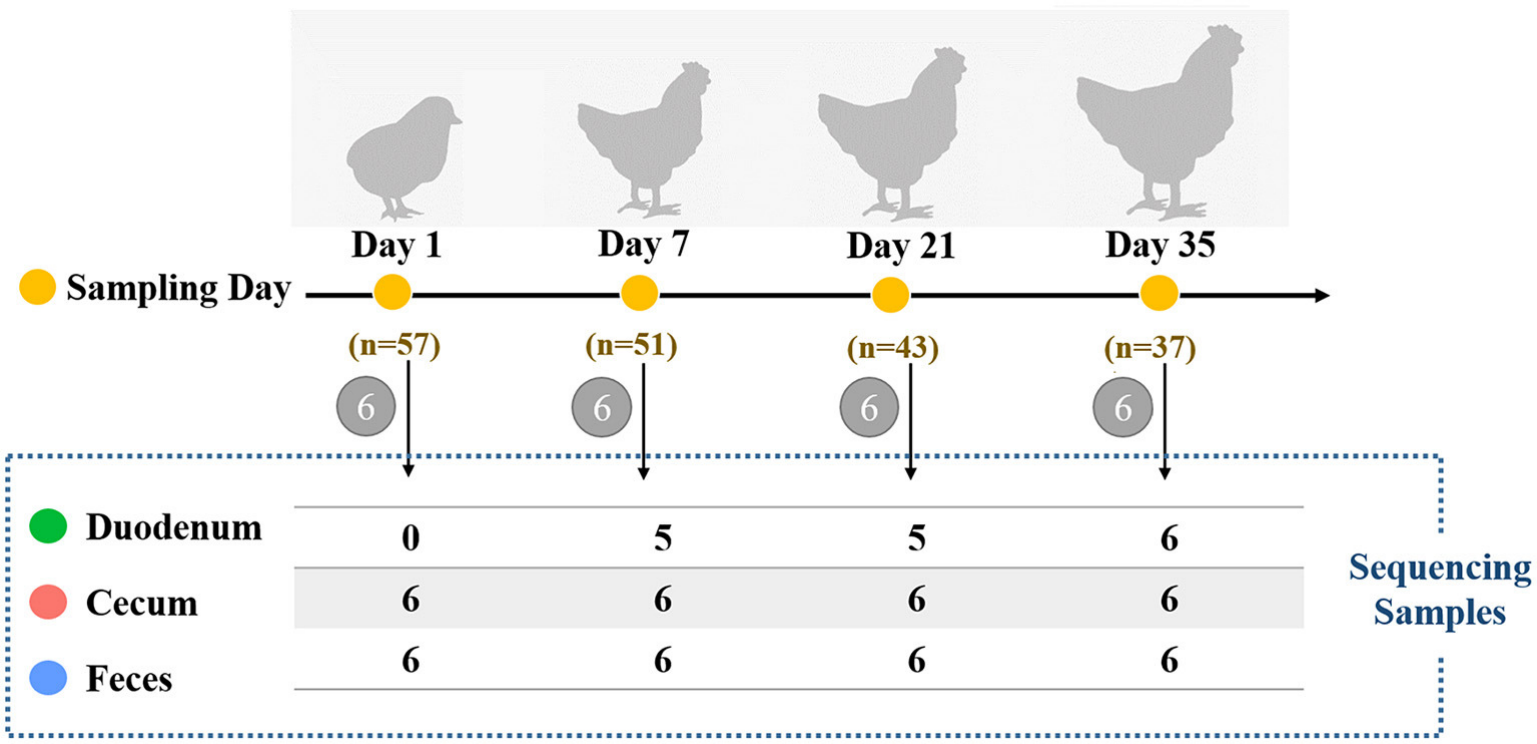
Schematic of the study design for assessing the influence of different timepoints or gut sections on chicken microbiota. Each sampling day is depicted in orange circles, and the number of birds at different timepoints is also shown. Six chickens were randomly selected for sample collection of the duodenum, cecum, and feces of each bird. The number of gut samples with sufficient sample amount for 16S rRNA sequencing is shown in the dashed box.

Male Arbor Acres broilers (*n* = 57) from a single hatch were raised in individual cages at the Poultry Genetic Resource and Breeding Experimental Unit of China Agricultural University. Birds were provided with *ad libitum* access to water and fed with two soybean-corn diets (Supplementary Table 1) from 1 day post-hatching to 5 weeks old. No drugs, prebiotics, probiotics, and antibiotics were used during the experimental period. In addition, we didn't use any vaccine because vaccines can also have a profound effect on the gut microbiota. The body weight of each bird was measured weekly with an electronic scale (to the nearest 5 g). As shown in Figure 1, chickens were sampled at 1, 7, 21, and 35 days of age (six individuals per age). On each sampling day, fresh fecal samples from each bird were collected by laying sterile plastic plates on the cage floor, and the droppings were collected as soon as excreta were discharged. The middle of the feces was collected to avoid environmental contamination. Birds were then euthanized by cervical dislocation followed by decapitation. Both the digesta and mucosa were sampled based on the consideration that the microbes from both sources may contribute to host interactions with respect to nutrient metabolism and immunity (24). The details of the collection of duodenal and cecal samples have previously been described by Yan et al. (25). In the duodenum, 6 samples at 1 day of age, 1 sample at 7 days of age and 1 sample at 21 days of age were excluded due to insufficient sample amount. A total of 64 intestinal samples remained for further DNA extraction and 16S rRNA gene sequencing. All samples were stored at−80 °C immediately after sample collection.

### DNA Extractions and 16S rRNA Gene Sequencing

Microbial DNA was extracted by using a QIAamp Stool Minikit (Qiagen, D4015-01, Hilden, Germany) according to the manufacturer's recommendations. The completeness of the DNA extract was checked by 1% agarose gel electrophoresis, and the final DNA concentration and purification were determined using a Nanodrop instrument (Thermo Fisher Scientific, Waltham, MA, USA).

PCR (polymerase chain reaction) amplification of the V4 region of the 16S rRNA gene was performed using the forward primer 515F (GTGYCAGCMGCCGCGGTAA) and the reverse primer 806R (GGACTACHVGGGTWTCTAAT). PCR was performed using ABI GeneAmp® 9700 (Applied Biosystems, Foster, CA, USA), and the reaction volume contained 4 μl 5× *TransStart* FastPfu buffer, 2 μl 2.5 mM dNTPs, 0.8 μl 5 μM forward primer, 0.8 μl 5 μM reverse primer, 0.4 μl *TransStart* FastPfu DNA polymerase, 0.2 μl BSA, 10 ng template DNA and ddH_2_O up to 20 μl. The PCR program was as follows: 95°C for 3 min, 27 cycles of 95°C for 30 s, 55°C for 30 s and 72°C for 45 s with a final extension of 72°C for 10 min (26).

The PCR product was extracted from a 2% agarose gel and purified using the AxyPrep DNA Gel Extraction Kit (Axygen Biosciences, Union City, CA, USA) according to the manufacturer's instructions and quantified using a Quantus™ Fluorometer (Promega, USA). After quantification, equimolar amounts of PCR products were pooled for paired-end sequencing, performed on the Illumina MiSeq PE300 platform according to the standard protocols by Majorbio Bio-Pharm Technology Co., Ltd. (Shanghai, China).

### Analysis of 16S rRNA Sequencing Data

The raw 16S rRNA gene sequencing reads were quality-filtered by fastp (ver 0.20.0) with default parameters (27) and merged by FLASH (ver 1.2.11) (28) according to the following criteria: (a) the 300 bp reads were truncated at any site receiving an average quality score of <20 over a 50-bp sliding window, and the truncated reads shorter than 50 bp were discarded. Reads containing ambiguous characters were also discarded; (b) only overlapping sequences longer than 10 bp were assembled according to their overlapping sequence. The maximum mismatch ratio of the overlapping region was 0.2, and unassembled reads were discarded; and (c) the number of primer mismatches was <2.

The resultant data were clustered by UPARSE (ver 7.1) to harvest operational taxonomic units (OTUs) with identities of >97% and filter chimera from the dataset. Sequencing data were then mapped to the Silva database (Release132) by RDP Classifier (ver 2.2) (29) using a confidence threshold of 0.7 (30). The singleton OTUs were discarded because they were generated mainly by sequencing errors.

### Characterizing the Spatial and Temporal Changes of the Gut Microbiota

An OTU count matrix was used to calculate the microbial diversity. The Shannon index and Simpson index were calculated to describe the community diversity and evenness of the gut microbial community using the vegan package (31) in R project (ver 4.0.2). To compare the differences in the alpha diversity index among groups, pairwise comparisons were conducted with the Wilcoxon rank-sum test. Principal coordinate analysis (PCoA) was conducted based on Bray–Curtis dissimilarities. The different groups were statistically compared through analysis of similarity (ANOSIM) with 999 permutations in the vegan package. The dynamics of the GIT microbiota at the phylum, family, and genus levels were presented in the form of alluvial diagrams and stacked histograms, respectively. A union set of genera with a mean relative abundance >2% in each gut section and timepoint was calculated. Linear discriminant analysis effect size (LEfSe) was performed to identify the bacteria enriched in different gut sections and different timepoints (32). The differences in features were identified at genus. The LEfSe analysis conditions were as follows: (1) the alpha value for the factorial Kruskal-Wallis test among classes was <0.05; (2) the alpha value for the pairwise Wilcoxon rank-sum test among subclasses was <0.05; (3) the threshold on the logarithmic LDA score for discriminative features was <4.0; and (4) multiclass analysis was set as all-against-all.

## Results

### Characterization of Host Phenotypes and Sequencing Output

The body weight of birds from hatching to 5 weeks of age was visualized in Supplementary Figure 1. Body weight increased rapidly from 14 days of age to 35 days of age and reached an average of 1,941.23 ± 224.37 g at the end of the trial.

A total of 64 samples collected from 1 day post hatching to market age (35 days of age) were analyzed to characterize the temporal and spatial dynamics of the gut microbiota (Figure 1). A total of 3,378,731 quality-filtered sequences were generated with an average of 52,793 reads per sample (Supplementary Table 2). These sequences were clustered into 1,057 OTUs and subsequently classified into 22 phyla, 37 classes, 100 orders, 178 families, 406 genera, and 580 species.

### Diversity and Composition of the Gut Microbiota

**Figure 2:**
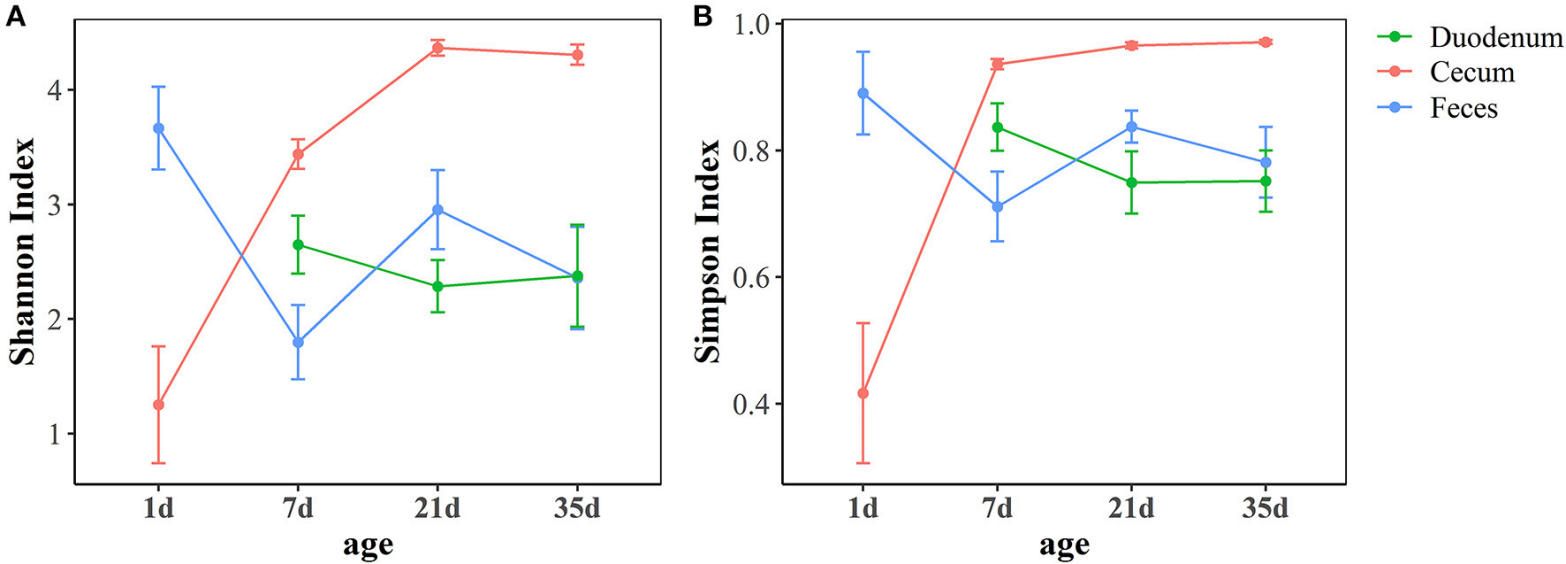
Age-related dynamics of alpha diversity measurements based on the Shannon index **(A)** and Simpson index **(B)** across three intestinal segments. The center point indicates the mean value in the corresponding group, and the data are expressed as the mean ± SE.

As shown in Figure 2, the Shannon index and Simpson index, which represent community richness and evenness, respectively, showed the same trend in different gut sections. In the feces, a high community diversity was exhibited at 1 day of age compared with the community diversity of other ages in this study. The high community diversity dropped dramatically at 7 days of age and increased at 21 days of age. Although, the community diversity of the fecal microbiota decreased at 35 days of age, no significant difference was observed compared with 21 days of age (*p* > 0.05, Supplementary Table 3). In the duodenum, the community diversity at 7 days of age was the highest and decreased at 21 days of age, while no significant changes were found between these two ages (*p* > 0.05, Supplementary Table 3). The results of the cecum demonstrated that the community diversity increased over time, reached the highest level at 21 days of age and then stabilized (Supplementary Table 3). Moreover, the cecum had higher community diversity than the other two sample types after 1 day of age (*p* < 0.05, Supplementary Table 4).

**Figure 3:**
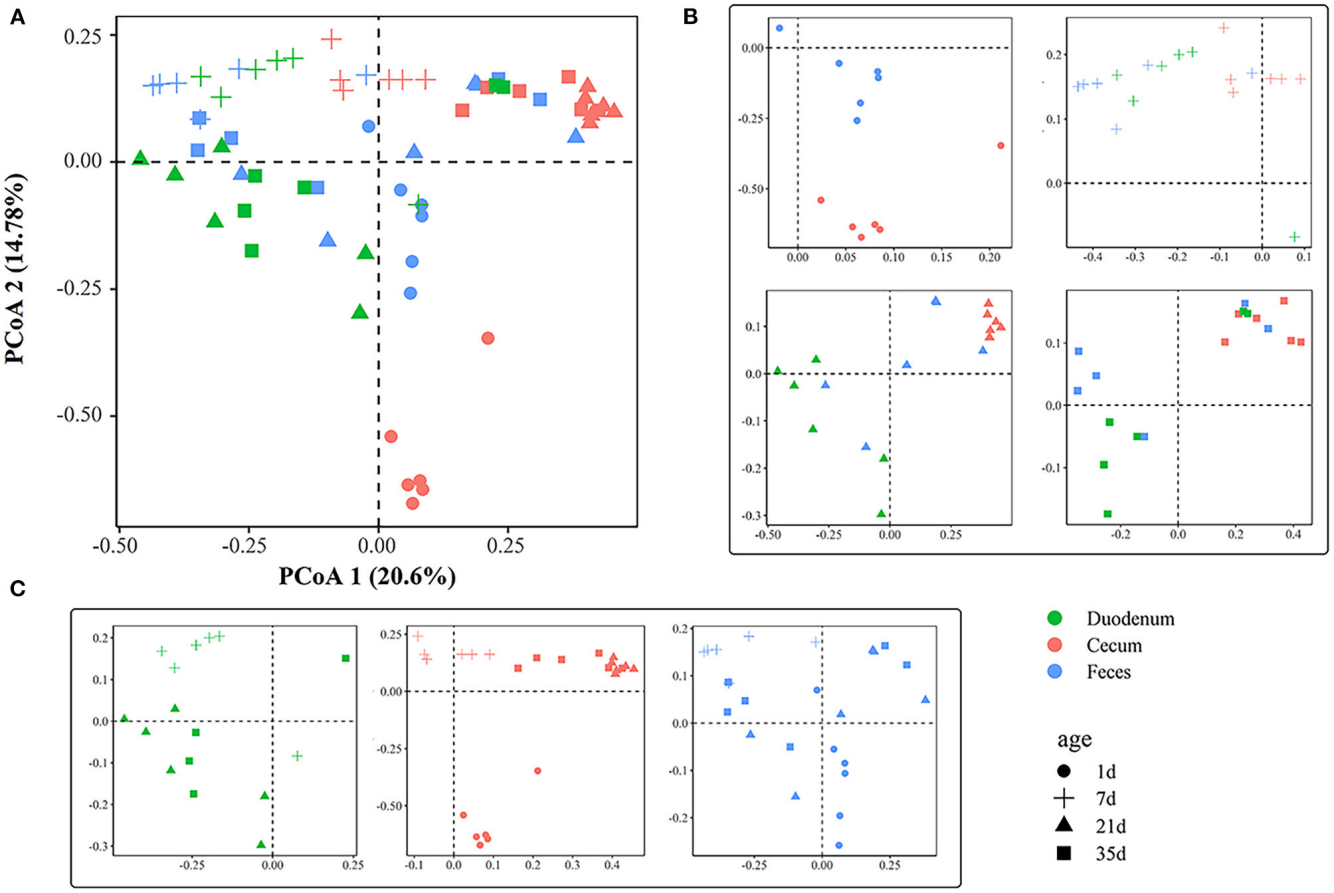
Principal coordinate analysis plot generated using OTU metrics based on Bray-Curtis dissimilarities. **(A)** Principal coordinate analysis plot of all samples according to age and gut sites. Each point represents a sample. **(B)** Principal coordinate analysis plots across gut sites. **(C)** Principal coordinate analysis plots across timepoints.

The PCoA plot showed an obvious difference among different gut sections at 1, 7, 21, and 35 days of age (Figures 3A,B), and ANOSIM confirmed this separation (*p* < 0.05, Supplementary Table 5) except between the duodenal and fecal samples at 7 days of age (*p* > 0.05, Supplementary Table 5). The microbial community structure exhibited clear differences among ages (Figure 3C). In the cecum, samples were clustered at 1, 7, 21, and 35 days of age (*R* > 0.79, *p* < 0.01, Supplementary Table 6). The gut microbiota of the feces was significantly divergent among 1, 7, and 21 days of age (*p* < 0.01, Supplementary Table 6). In the duodenum and feces, samples at 21 days of age were indistinguishable from those at 35 days of age. The results from ANOSIM showed that the duodenal microbial structure between 21 and 35 days of age was similar (*R* < 0.15, *p* > 0.05, Supplementary Table 6). A similarity of microbial communities was also found in the feces between the two ages.

**Figure 4:**
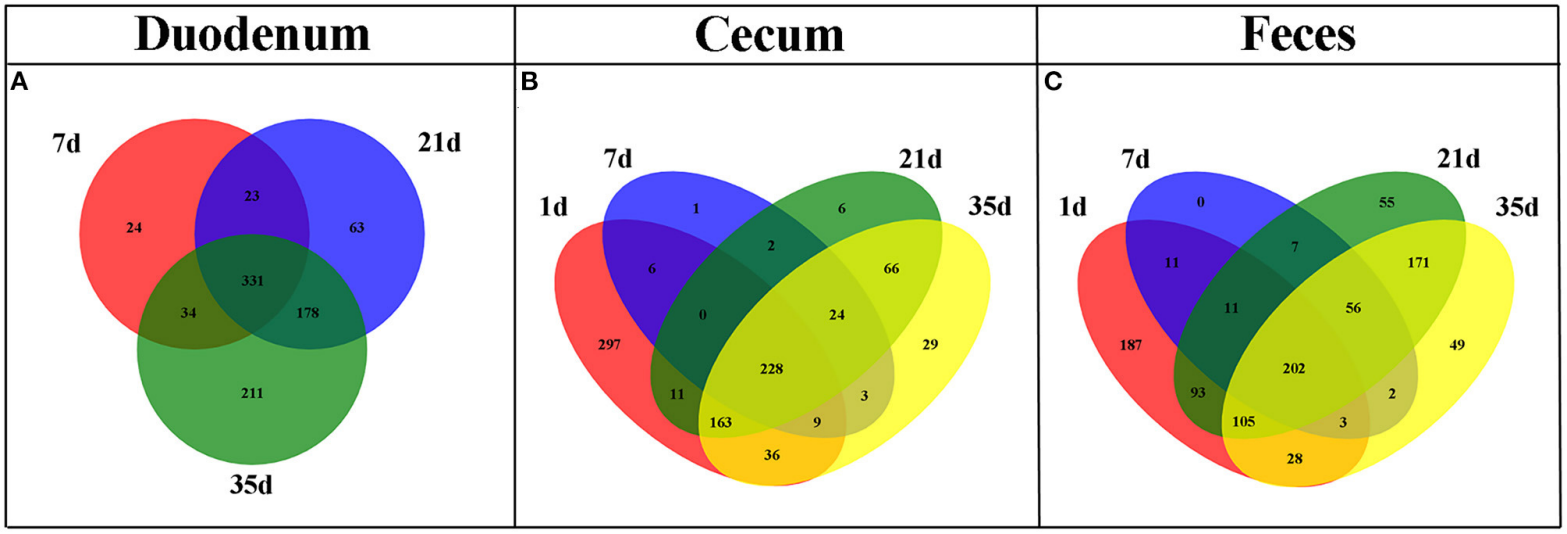
Venn diagram illustrating core OTUs across different timepoints in duodenal **(A)**, cecal **(B)**, and fecal **(C)** samples.

The shared taxa at all timepoints in the duodenum, cecum and feces were deemed to be core bacterial microbiota and were shown by a Venn diagram. We observed that 331 OTUs were shared across all timepoints in the duodenum, 228 in the cecum and 202 in the feces (Figure 4). These OTUs represented high proportions of sequences in all subgroups except cecal samples at 1 day of age (Supplementary Figure 2), indicating that the most abundant members detected in these groups belonged to the core microbiota. Moreover, the number of common OTUs between 21 and 35 days of age at different sites was higher than the number of common OTUs in the other groups (Figure 4).

**Figure 5:**
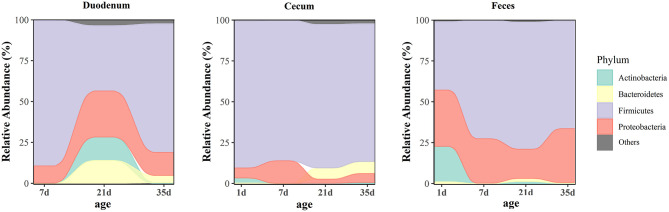
Age-related dynamics of the top four predominant microbial phyla grouped by gut sites. The average abundance of each group is shown in an alluvial plot.

**Figure 6:**
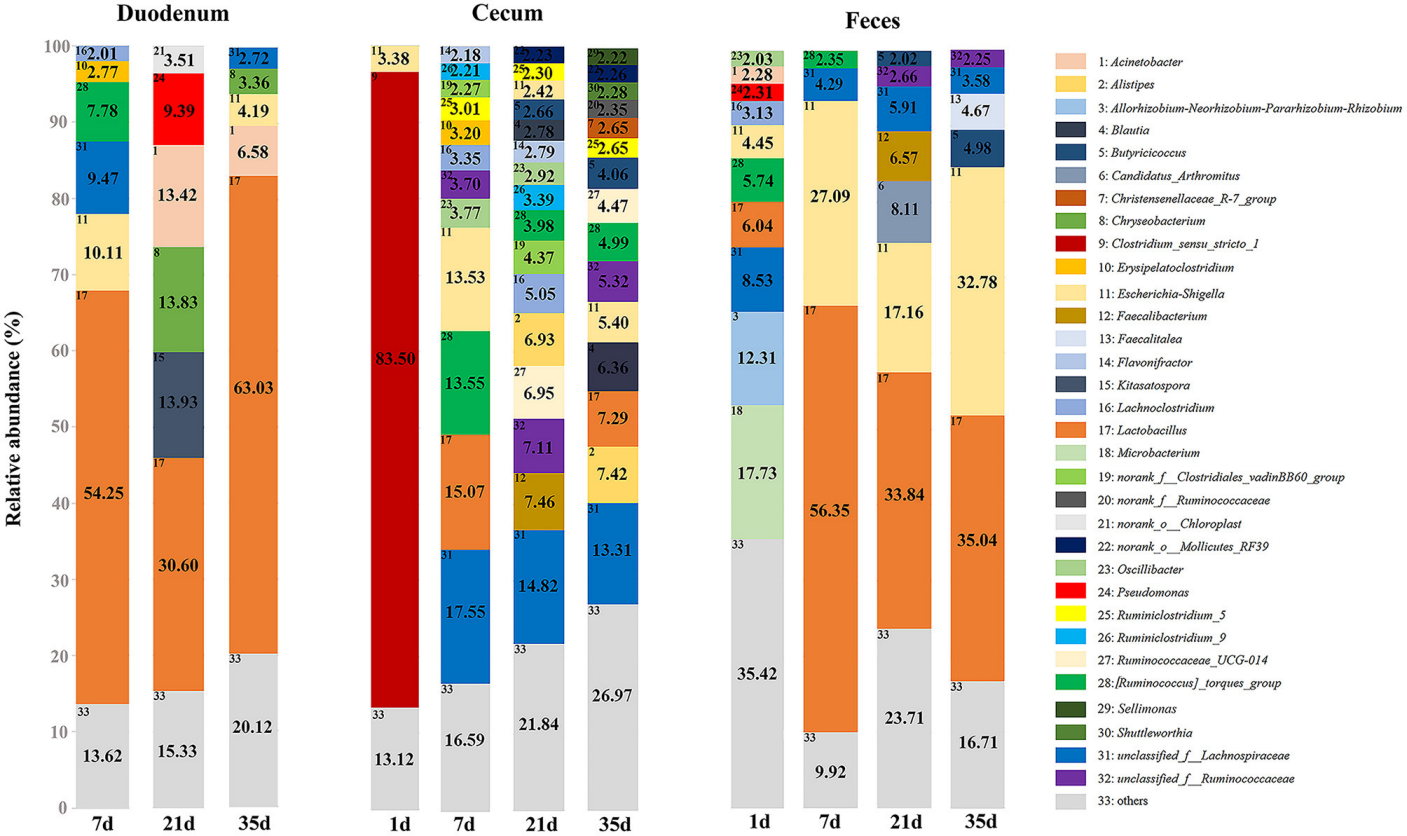
Relative abundance of predominant genera of groups in different gut sites. Only the genera with an average abundance of over 2% in each group are shown with annotation information.

At the phylum level, microbiota displayed different abundances with respect to age (Figure 5 and Supplementary Table 7). The three gut segments had similar dominant phyla, in which Firmicutes, Proteobacteria, Actinobacteria, and Bacteroidetes were the top four phyla. Firmicutes was the most abundant phylum, followed by Proteobacteria, across each age group, and these phyla accounted for more than 90% of the total sequences. Lactobacillaceae was the most abundant family in the duodenum and feces except 1 day of age in the feces. The cecum became populated by family Clostridiaceae immediately after hatching. A week later, the members of family Lachnospiraceae and Ruminococcaceae became predominant (Supplementary Figure 3 and Supplementary Table 8). Among the top 32 genera with over 2% abundance, 22 belonged to the phylum Firmicutes. The distribution and dynamics of relative abundance among different bacterial genera were shown in Figure 6. In the duodenum and feces, the genus *Lactobacillus* dominated the bacterial community, except for fecal samples at 1 day of age. *Escherichia-Shigella* accounted for a large proportion of the feces (17.16–32.78%) after 1 day of age. Interestingly, *Clostridium_sensu_stricto_1* accounted for 83.50% of the total sequences in the cecum at 1 day of age but decreased substantially thereafter (Supplementary Table 9).

### The Segment- and Age-Related Bacteria

**Figure 7:**
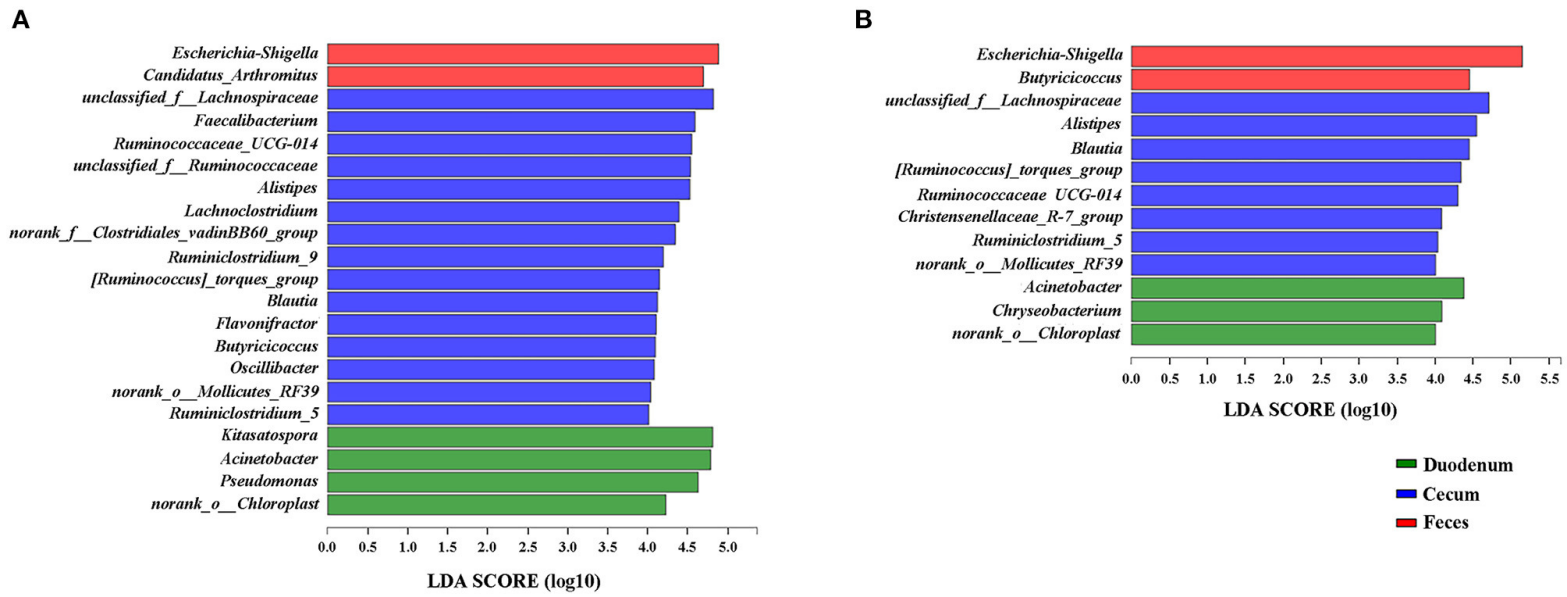
LEfSe results for the duodenal, cecal, and fecal microbiota at 21 **(A)** and 35 **(B)** days of age. Only LDA scores above 4 are shown.

Because the microbial diversity and composition of samples were similar between 21 and 35 days of age, the genera identified to be significantly representative of each gut section were taken by LEfSe at these two timepoints (Figure 7). We identified *Escherichia-Shigella* as a biomarker of 21 and 35-day-old broilers (LDA effect size > 4) in the feces and *Acinetobacter* in the duodenum. Five genera, including *Alistipes, Blautia, Ruminiclostridium_5, Ruminococcaceae_UCG-014*, and *[Ruminococcus]_torques_group*, were significantly enriched in the cecum at both 21 and 35 days of age. Interestingly, *Butyricoccus* was a significantly representative genus of the cecum at 21 days of age; however, *Butyricoccus* was a biomarker of the feces at 35 days of age. Furthermore, the genera identified to be representative microbiota of each time point in the duodenum, cecum and feces were also shown in Supplementary Figure 4. *Clostridium_sensu_stricto_1* was the most significant biomarker of 1 day of age in the cecum.

### The Dynamics of Predominant and Segment-Related Bacteria

**Figure 8:**
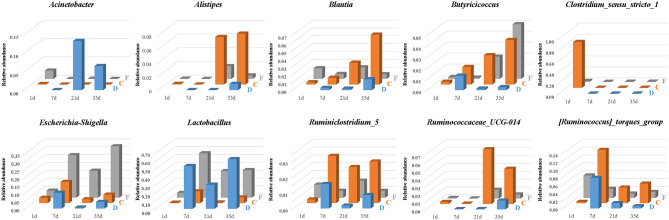
The temporal and spatial dynamics of predominant and segment-related genera in the duodenum (D), cecum (C), and feces (F). The average abundance of each group is presented in a bar graph.

The temporal and spatial dynamics of the predominant and segment-related genera were shown in Figure 8. The genera *Lactobacillus* and *Escherichia-Shigella* persisted throughout life, and their colonization followed an age-specific pattern. *Lactobacillus* was listed as a numerically dominant genus in the duodenum and feces but presented much lower abundance in cecal samples. Similar dynamic changes of *Lactobacillus* among ages were detected in three gut segments. The relative abundance of *Lactobacillus* was low on the first day, substantially increased until 7 days of age, declined at 21 days of age, and revived thereafter. The age-associated dynamic trend of *Escherichia-Shigella* coincided with that of *Lactobacillus*. *Escherichia-Shigella* was found to have a higher abundance in the feces than in cecal and duodenal samples.

Microbial biomarkers for the cecum included *Clostridium_sensu_stricto_1, Alistipes, Blautia* and three genera from the family Ruminococcaceae *(Ruminiclostridium_5, Ruminococcaceae_UCG-014* and *[Ruminococcus]_torques_group*), whose abundances were altered with age (Figure 8). The relative abundance of *Clostridium_sensu_stricto_1* in the cecum was 83.50% at 1 day of age and then sharply decreased to <0.01% at 7 days of age. In addition, *Butyricicoccus* was present in the cecum and feces with an increasing relative abundance across age. In the duodenum, *Butyricicoccus* was observed with the highest abundance at 7 days of age.

## Discussion

The chicken gut microbiome is considered to play important roles in host nutrition absorption, development of immunity, and disease resistance and has received growing attention (33, 34). Gaining an insight into how the microbiota changes over time and the differences among gut segments may help to better comprehend the microbial ecology of the chicken gut and further improve chicken nutrition, disease resistance, and productivity. We herein compared the microbial diversity and composition of the duodenum, cecum and feces from 1 day post-hatching to 35 days of age in broilers.

The diversity of the cecal microbial community was higher than the diversity of other gut segments after 7 days of age, and similar findings were reported by Wen et al. (5) and Xiao et al. (35). Community richness of the cecum increased rapidly during the early growth stage and remained relatively constant, which was in accordance with previous study in chicken (12). We confirmed that microbiota in the cecum became progressively divergent with age and was more diverse and complex than the microbiota of other gut segments (36). Inconsistent with the cecum, high community diversity in feces at the beginning of life was in agreement with findings in broilers (37), indicating a rapid intake of environmental organisms after birth. In the duodenum, no significant changes were found in alpha diversity across time. It can be inferred that the patterns of gut microbial diversity differed with the intestinal segment in our study.

Beta diversity displayed distinct clusters separating the microbiota of subgroups, which supported the importance of age (38) and gut sites (39) in affecting the gut microbiome. The gut microbiota extracted from samples collected at 21 and 35 days of age clustered in close proximity and exhibited similar community diversity and composition. Age-associated changes in the gut microbiota may reveal that the colonization of microbiota is dynamic, and the succession of microorganisms can be affected by diet, defense against disease and interaction with the host or one another; then, the microbial community becomes more diverse until it reaches a state of relative equilibrium (6).

Based on the findings in this study and clues from previous reports, we proved that the gut microbiota of broilers was dominated by the phyla Firmicutes and Proteobacteria in the duodenum, cecum and feces during different growth stages (12, 15). The succession of communities was different in each gut segment, and the cecal microbiota was initially formed by predominantly *Clostridium_sensu_stricto_1* which diversified over time to contain dominant representatives of family Lachnospiraceae and Ruminococcaceae, with smaller numbers of other taxonomies (13, 40). Microbial communities in chickens, as previous studies have shown, are initially dominated by members of the families Enterobacteriaceae and Clostridiaceae (41, 42), which serve as founding species for chicken gut microbial communities. *Clostridium_sensu_stricto_1*, belonging to the family Clostridiaceae, exhibited significantly higher abundance at 1 day of age than at any other age due to its ubiquity in the environment, whereas its abundance decreased sharply with the rapid growth of the gastrointestinal tract in chickens (12, 40). We hypothesized that *Clostridium_sensu_stricto_1* acted as a member of the founding species and that it decreased with the colonization of other microorganisms. Furthermore, *Clostridium_sensu_stricto_1* has been reported to be correlated with necrotic enteritis (43), and perhaps the lack of a sound immune system and acute environmental susceptibility in 1-day-old broilers resulted in the high abundance.

At the genus level, we focused on the succession of several predominant genera and found that *Lactobacillus* featured more prominently throughout most of the timepoints in chicken gut segments other than the cecum (44). In addition, *Lactobacillus* has been reported to play a prominent role in improving chicken feed efficiency (45), and bacteria of the genus were recognized as an important candidate for probiotics (46, 47). The dynamic succession of *Lactobacillus* was expected as previous studies conducted on developing chicken microbiome and showed that *Lactobacillus* initially accounted for an average of low abundance, which maintained a relatively high abundance, fluctuating thereafter in the duodenum (48) and feces (12). *Escherichia-Shigella* is another genus that is universally found in chicken GIT and feces (45, 49). *Escherichia-Shigella* belongs to the family Enterobacteriaceae and is generally found in higher proportions in broiler feces than in cecal samples (50). *Escherichia-Shigella* has been recognized to be negatively correlated with growth and fat digestibility in broilers (51). Moderate antibiotics (52) and supplementation with organic acids (53) are capable of inhibiting *Escherichia-Shigella* and promoting the growth performance of poultry. The increasing concentration of short-chain fatty acids in the broiler cecum has been suggested to be responsible for the decline of Enterobacteriaceae during growth (54).

The cecum is a complex ecosystem that includes a highly varied microbiome, within which the cecum functions as a fermenter for decomposing the most indigestible residues to generate short-chain fatty acids (SCFAs) (19, 55). SCFAs are absorbed transepithelially to supply energy requirements for chickens (56). The production of SCFAs in the chicken gut has been shown to be able to act as an indicator of the presence of bacterial groups that are beneficial to health and growth performance (57, 58).

A number of SCFA producers which belong to the family Ruminococcaceae, including *Ruminiclostridium_5, Ruminococcaceae_UCG-014*, and *[Ruminococcus]_torques_group*, were significantly enriched in the cecum. These bacteria are considered as dominant players in the degradation of diverse polysaccharides and fibers (59, 60). *Ruminiclostridium_5* and *[Ruminococcus]_torques_group* were found to be related to fat deposition (52, 61), while *Ruminococcaceae UCG-014* was linked to the maintenance of gut health and was able to degrade diverse cellulose and hemicellulose with enzymatic capability (62). The genera *Blautia* and *Butyricicoccus* were also recognized as cecal biomarkers in our study. Bacteria in the *Blautia* genus, producing acetic acid via acetyl-CoA from pyruvate and the Wood-Ljungdahl pathway by fermenting glucose and indigestible diet fiber (63–65), have been reported to be associated with obesity (66). *Butyricicoccus* is a potential active component of probiotic formulations (67) and a producer of SCFAs, especially butyrate (68). Similar to previous study in the broiler cecum, the proportion of genus *Butyricicoccus* showed highly positive correlations with age, corresponding to the growth and development of the body (69).

Different segments of the GIT vary immensely in oxygen content (70), and the aerobic conditions in the duodenum afford an opportunity for the growth of aerobic bacteria. *Acinetobacter*, a strictly aerobic bacterium, is one of the genera represented with high abundance in the duodenum (71).

In summary, our study profiled the microbial communities of the duodenum, cecum, and feces, and we confirmed that the gut microbiota was altered with growth and different gut segments. The community diversity of the cecum increased rapidly over time and gradually reached a relatively stable state. LEfSe analysis further identified several genera as distinct gut segment biomarkers, notably associating the cecum with the elevated occurrence of SCFA-producing bacteria. In addition, the temporal and spatial dynamics of several predominant and segment-related genera were described, which could lead to a greater understanding of the microbial ecology of the chicken gut.

## Data Availability Statement

The datasets presented in this study can be found in online repositories. The names of the repository/repositories and accession number can be found below: NCBI Sequence Read Archive under BioProject ID PRJNA731064.

## Ethics Statement

The complete procedure was performed following recommendations for the regulations and guidelines established by the Animal Care and Use Committee of China Agricultural University (permit number: SYXK 2015-0028).

## Author Contributions

NY and CW designed the study. QZ, XL, WY, CS, and JL collected the samples. QZ and FL analyzed the data and wrote the manuscript. CW assisted in data analyzing. NY and CW contributed to the revisions. All authors read and approved the final manuscript.

## Conflict of Interest

The authors declare that the research was conducted in the absence of any commercial or financial relationships that could be construed as a potential conflict of interest.

## Publisher's Note

All claims expressed in this article are solely those of the authors and do not necessarily represent those of their affiliated organizations, or those of the publisher, the editors and the reviewers. Any product that may be evaluated in this article, or claim that may be made by its manufacturer, is not guaranteed or endorsed by the publisher.

## Supplementary Material

Supplementary Figure 1 Change curves of body weight from hatching to market age of broilers.

Supplementary Figure 2 The percentage of core OTUs and sequences represented by these OTUs in duodenal **(A)**, cecal **(B)**, and fecal **(C)** samples.

Supplementary Figure 3 Relative abundance of the top 14 predominant families of groups in different gut sites. Only the families with an average abundance of over 10% in each group are shown with annotation information.

Supplementary Figure 4 LEfSe results for different timepoints in duodenal **(A)**, cecal **(B)**, and fecal **(C)** samples. Only LDA scores above 4 are shown.

Supplementary Table 1 Ingredients and nutrient composition of diets.

Supplementary Table 2 Summary statistics of 16S rRNA gene sequencing.

Supplementary Table 3 Wilcoxon rank-sum test based on the Shannon index and Simpson index across timepoints.

Supplementary Table 4 Wilcoxon rank-sum test based on the Shannon index and Simpson index across gut sections.

Supplementary Table 5 Analysis of similarity was used to determine chicken gut microbiome dissimilarities between different gut sections at four timepoints based on Bray-Curtis distances.

Supplementary Table 6 Analysis of similarity was used to determine chicken gut microbiome dissimilarities between different timepoints in three gut sections based on Bray-Curtis distances.

Supplementary Table 7 The average abundance of phyla among different intestinal sections at all timepoints.

Supplementary Table 8 The average abundance of families among different intestinal sections at all time points.

Supplementary Table 9 The average abundance of genera among different intestinal sections at all time points.
