# Supplementary figures and images for: The Spatial and Temporal Characterization of Gut Microbiota in Broilers
Source: Front Vet Sci. 2021 Aug 30;8:712226. doi: 10.3389/fvets.2021.712226 (PMC8435590; doi:10.3389/fvets.2021.712226)

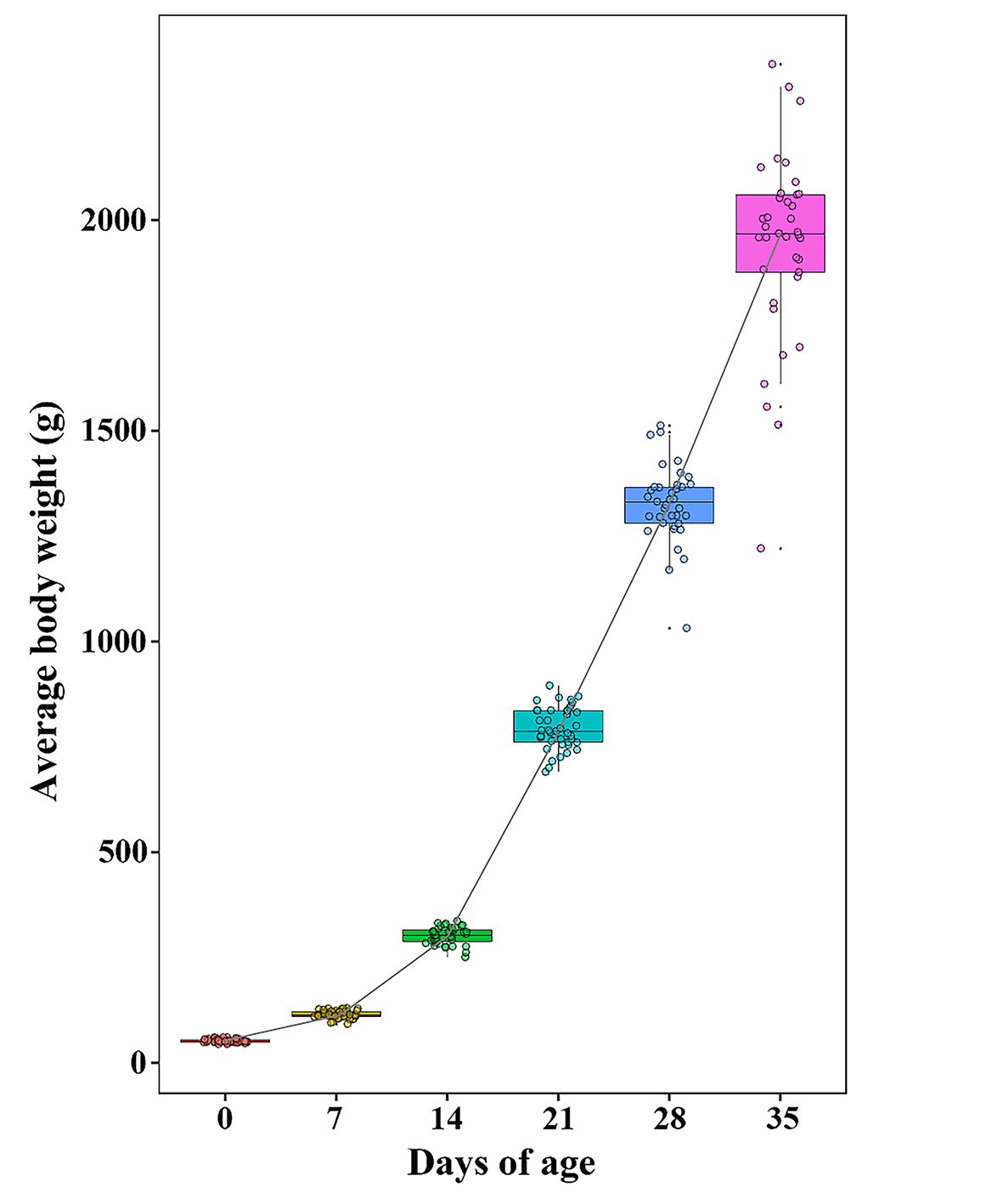

Supplement: Supplementary Figure 1 — Change curves of body weight from hatching to market age of broilers. [file Image_1.TIF]

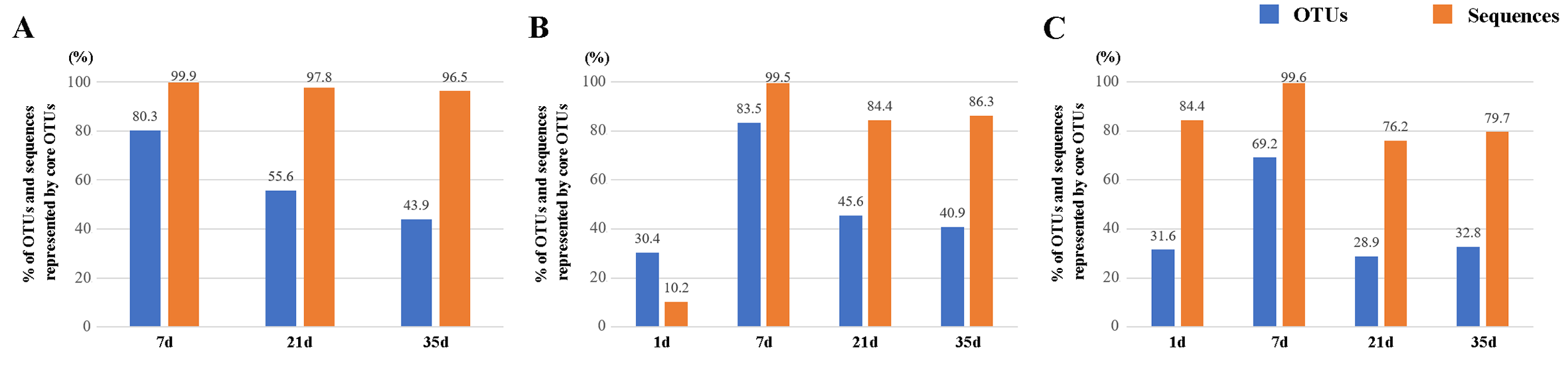

Supplement: Supplementary Figure 2 — The percentage of core OTUs and sequences represented by these OTUs in duodenal (A), cecal (B), and fecal (C) samples. [file Image_2.TIF]

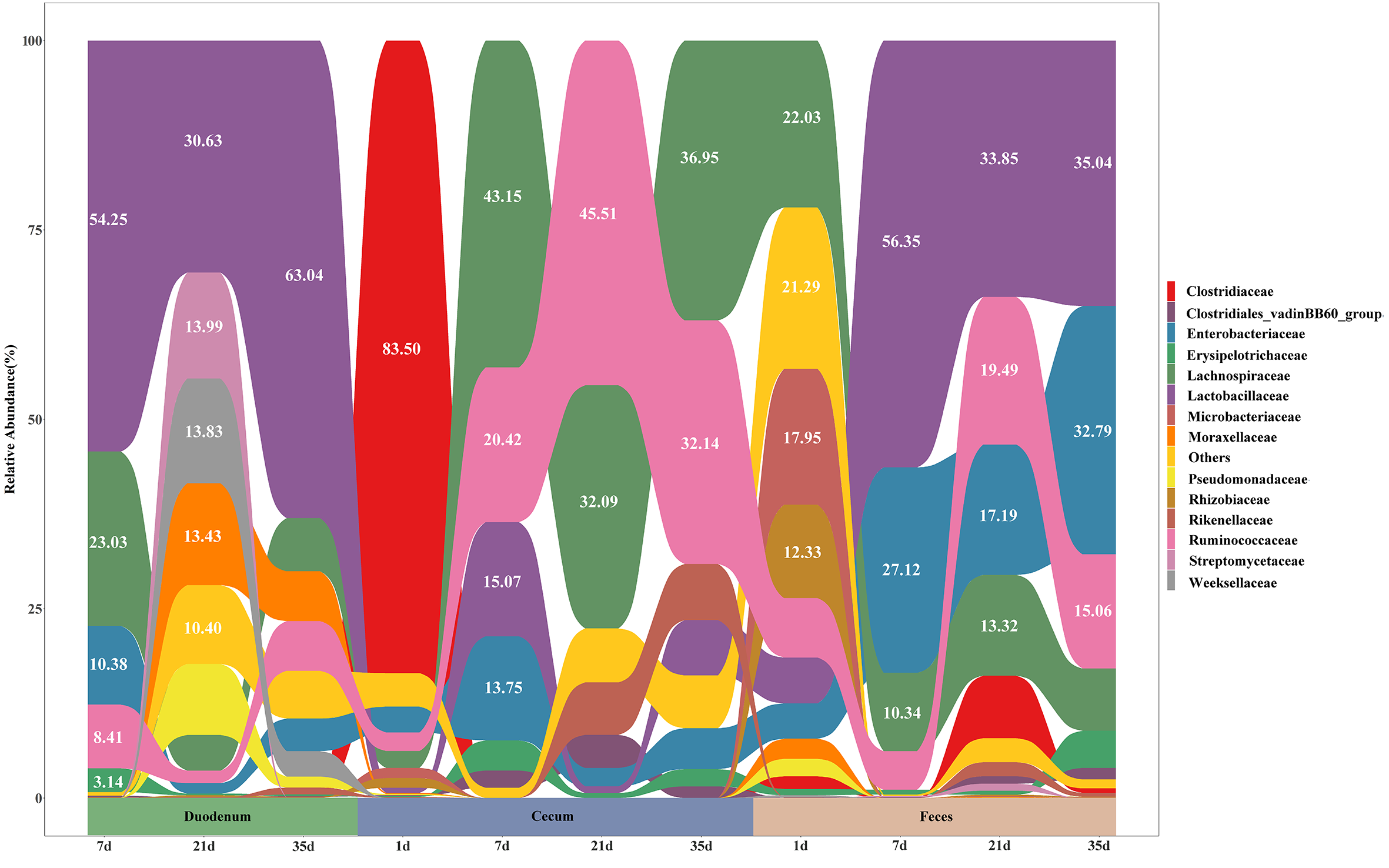

Supplement: Supplementary Figure 3 — Relative abundance of the top 14 predominant families of groups in different gut sites. Only the families with an average abundance of over 10% in each group are shown with annotation information. [file Image_3.TIF]

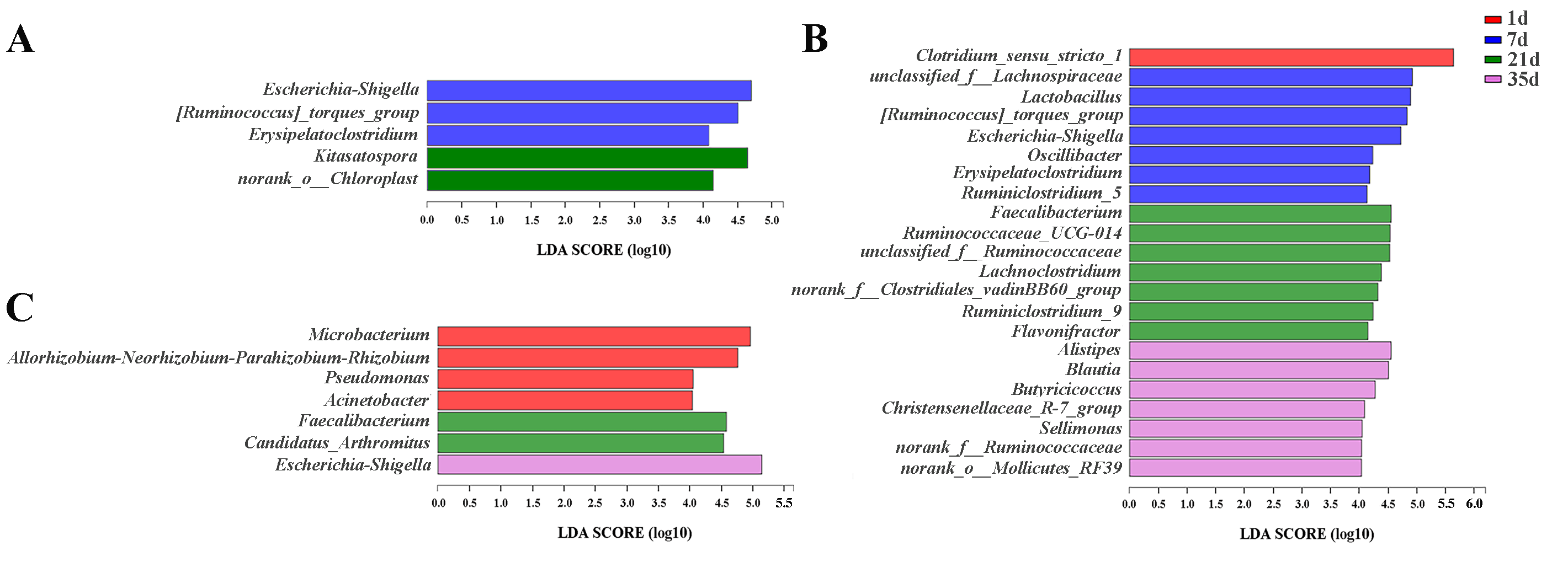

Supplement: Supplementary Figure 4 — LEfSe results for different timepoints in duodenal (A), cecal (B), and fecal (C) samples. Only LDA scores above 4 are shown. [file Image_4.TIF]
